# Could you have said no? A mixed-methods investigation of consent to HIV tests in four African countries

**DOI:** 10.7448/IAS.17.1.18898

**Published:** 2014-03-17

**Authors:** Carla Makhlouf Obermeyer, Cairn Verhulst, Khalil Asmar, for the MATCH study group

**Affiliations:** 1 Center for Research on Population and Health, Faculty of Health Sciences, American University of Beirut, Beirut, Lebanon; 2 Independent Consultant, Dakar, Senegal; 3 Department of Epidemiology and Population Health, Faculty of Health Sciences, American University of Beirut, Beirut, Lebanon

**Keywords:** Consent, HIV, testing, ethics, sub-Saharan Africa, qualitative

## Abstract

**Introduction:**

Although most studies report high frequencies of consent to HIV tests, critics argue that clients are subject to pressure, that acceptors later indicate they could not have refused, and that provider-initiated HIV testing raises serious ethical issues. We examine the meaning of consent and why clients think they could not have refused.

**Methods:**

Clients in Burkina Faso, Kenya, Malawi and Uganda were asked about consenting to HIV tests, whether they thought they could have refused and why. Textual responses were analyzed using qualitative and statistical methods.

**Results:**

Among 926 respondents, 77% reported they could not have said no, but in fact, 60% actively consented to test, 24% had no objection and only 7% tested without consent. There were few significant associations between categories of consent and their covariates.

**Conclusions:**

Retrospectively asking clients if they could have refused to test for HIV overestimates coercion. Triangulating qualitative and quantitative data suggests a considerable degree of agency.

## Introduction

One of the most contentious issues in debates around the scale-up of HIV testing is whether informed consent can be ensured when testing is routinized [1–4]. Recent reviews have shown the trade-offs of different approaches to testing [5, 6] and an analysis of testing policies in sub-Saharan Africa has highlighted complicated ethical dilemmas regarding consent and confidentiality when HIV policy is put into practice “on the ground” [7]. Observers have expressed concern that routine testing may be perceived as mandatory, and that some individuals who appear to consent have, in fact, been subjected to various degrees of coercion [8–10].

There is a vast literature on informed consent, and it is mostly focused on two of the three elements that are deemed to be essential, namely information and competence to make a choice. The third element, voluntariness, has received less attention, but there are concerns that participants, especially in the developing world, frequently believe they are not free to refuse or withdraw [11, 12]. Regarding HIV, an often-cited study [13] reported that among 56 antenatal clinic attenders in South Africa who consented to participate in a research project, 88% later reported feeling pressured. Since then, the option to refuse has been highlighted, and researchers have tried to ascertain consent by asking clients retrospectively whether they thought they could have refused to test for HIV. The evidence from surveys seems to substantiate concerns about coercion. In Botswana, 93% of 1268 participants consented to test, but 68% believed they could not refuse [14]. In rural Kenya, virtually all 900 respondents accepted to test, but only 20% thought they could decline [15]. In a provider-initiated testing program in Toronto, 30% of 299 women said they did not believe they could decline to test [16]. And in Malawi, the majority of 18 antenatal clinic attenders perceived there was no option to refuse testing [8]. Such results have been attributed to clients’ dependence on providers for health services, their fears that their care would be jeopardized if they refused and, more generally, the power differential between patients and health professionals.

The discrepancy between explicit acceptance of testing and perceived option to refuse raises important questions regarding the optimal way to assess consent and what constitutes unacceptable pressure. Because voluntariness is a somewhat subjective notion, it cannot be fully captured by simple indicators, and efforts are needed to elicit individuals’ perceptions and experiences. But large surveys are constrained by the use of closed-ended questions, while studies that listen to clients’ voices tend to be small-scale, with limited generalizability. The MATCH (Multi-country African Testing and Counselling for HIV) study represents an effort to combine quantifiable and textual data about HIV testing on a relatively large sample in four African countries. Indicator measures based on closed-ended questions showed that the near totality of respondents reported consenting [17]. Additional data about the circumstances of testing were also collected, based on clients’ responses to open-ended questions. The availability of transcribed texts on over 900 respondents represents a special opportunity to measure voluntariness and gain insights into clients’ decisions to test.

The objectives of this analysis are to ascertain the extent of consent, based on clients’ reports about their experience; examine the reasons why some respondents thought they could not have refused; analyze the associations between consent, reasons and their covariates; and draw the implications for HIV testing programs and for definitions and measures of consent.

## Methods

The MATCH study was designed to investigate HIV testing in four countries. A cross-sectional survey of clients was conducted in 2008–09 in Burkina Faso, Kenya, Malawi and Uganda, at health facilities representing prevalent modes of testing and major providers of testing services. All respondents present at the selected facilities on the days of the survey, and who agreed to discuss their experience, were interviewed. Data were collected about respondents’ socio-demographic characteristics and their experience with HIV testing, including their descriptions of consenting to test. The study was cleared by the institutional review board of each of the four countries, and by the Ethics Review Committee of the World Health Organization. Details about the selection of facilities and respondents, response rates, ethical clearance and data collection have been previously described [17].

We used data from the consent module in the survey, specifically, responses to the closed-ended question: “Do you think you could have said no?”, and the open-ended question: “Why or why not?” Textual responses were recorded as close to verbatim as possible and transcribed. This analysis focuses on two outcome variables: the extent of consent based on clients’ textual responses, and clients’ reasons for thinking they could not refuse. Covariates that may have influenced outcome variables include age, gender, education and a wealth index based on assets and household amenities, previously described [17].

The analysis of textual data was conducted in NVivo 9.2. [18]. The transcribed texts were repeatedly reviewed to discern patterns in discourse about consent. Based on the themes in the texts, a four-way typology of consent was developed. A thorough review of each of the 926 responses was conducted by two authors (CMO, CV) in order to classify respondents into categories of consent, resolve differences and ensure consistency. The texts regarding reasons for not being able to refuse were similarly reviewed and recoded, the themes regrouped and the classification reviewed by two authors. In addition to software-based recoding and classifying, the texts were read to select illustrative quotes and to gain insights into decisions regarding testing.

The four-way consent variable and the grouped reasons why people thought they could not refuse were merged back into the quantitative data set. Using bivariate (Chi-square) and multinomial logistic regression analysis, we assessed their associations with covariates. We modelled the odds that a respondent was in the “No Objection” or “No Consent” category, respectively, compared with “Active Consent” as the reference category. We also modelled the likelihood of referring to health, decisions or providers as reasons why respondents reported they could not have said no. The multinomial logistic regression analysis adjusted for country using a fixed effect, and for clustering of responses at the interview facility. All statistical analyses were completed in Stata SE 10.1 [19].

## Results

A total of 2153 respondents interviewed at health facilities reported that they tested for HIV in 2007 or later. Respondents who reported testing at their own initiative (1217) skipped the consent module; other respondents were asked whether they thought they could have refused and why; 926 respondents provided responses to both questions. They were mostly young adults (46%, aged 25–34); 29% were HIV-positive; 69% were women; and 42% had secondary education. These characteristics are similar to those of the full sample, except that the full sample had a slightly lower percentage of women and higher percentage with secondary education (63% and 48% respectively).

Detailed sample characteristics are presented in Supplementary file S1.

In response to a closed-ended question, 95% of the 926 respondents reported that they agreed to test, 88% said they were told they had a choice to agree or refuse, 90% said that no one else was involved in their agreement to test and 93% thought it was important to be able to refuse to test. These frequencies suggest prior consideration of HIV testing and individual choice. Yet when asked if they thought they could have said no, only 23% said they could have refused. To better understand this inconsistency, we turn to the textual data transcribed from respondents’ statements.

### Consent based on respondents’ statements

Textual responses provide details, in respondents’ words, about the circumstances of testing, and represent a sound basis for assessing voluntariness. Respondents who referred to proactive decision-making, explicit consent, wanting to know their status, prior decision, or the importance of knowing HIV status were classified as having consented. Those who stated that they did not consent, did not know they were being tested, or perceived testing to be mandatory were classified as not having consented.

After reviewing textual responses, it became clear that a considerable number of respondents did not fit into this binary classification. Based on themes in the texts, two additional categories were created. The first, “No Objection,” groups respondents who had neither strong motivations to test nor clear reasons to refuse: they tested without prior deliberation, as a result of circumstances, usually in the course of health care, and while they did not initiate testing, they conveyed they were not opposed to it. The second category, “Ambivalent,” groups respondents who expressed conflicting desires and continuing doubts about the decision to test: they may have wanted to test but had reservations; may have known they had a right to refuse, but felt they did not want to exercise it; or found themselves in situations where they could not refuse. A third category, “Indeterminate,” was created for those respondents whose statements were unclear or too brief to be classified. Criteria used to classify respondents and illustrative quotes are presented in Table 1.

**Table 1:** Categories of consent to HIV testing

Category	Criteria	Illustrative excerpts from respondents’ statements
1. Actively consented	Wanted to test Made a prior decision Concerned about health and sees test as important Favourable to testing	I wanted to know my status I wanted to confirm my status I wanted the test; I wanted treatment Testing with partner I wanted to get help To be able to plan for my life I was ready for it
2. No objection to testing	No reasons to refuse testing Took the opportunity to test when offered Not overly concerned/somewhat nonchalant about testing No problem with recommendation to test	No reason to refuse Testing is part of the treatment I was referred It was the opportunity to do it Provider was friendly Test is free I was not worried about testing I was told that I have the choice I was not forced, I had the right to say no
3. Ambivalent about testing	Conflicting desires to test or not Previously consented, though not sure at time of test Statement includes both positive and negative points about testing Could theoretically refuse but felt they could not Not quite ready but accepted	Initially agreed; once you decide, you can’t refuse You can’t refuse those who treat you I did as I was told I feared the outcome, was not fully prepared to know I don’t know why I accepted, I was not myself
4. Did not consent	Did not know was being tested Was not given a choice Thought testing was mandatory	Test was mandatory I had no option to refuse Forced to test by provider I did not know I was testing for HIV
5. Indeterminate	Contradictory or unclear statement Insufficient information	

Table 2 shows the frequencies of the four-way consent classification for the 926 respondents. The majority of respondents (60%) actively consented to test, 24% had no objection, 4% were ambivalent, 7% did not consent and 5% were indeterminate. Bivariate associations of the new consent variable with covariates (Supplementary file S2) indicate that only HIV-positive status was significantly associated with consent. The statements of the 710 respondents (77% of the sample) who did not think they could have said no show that 67% had in fact actively consented, 9% had not consented and 21% had no objection or were ambivalent.

**Table 2:** Frequency of consent variable, by whether or not respondents thought they could have said no (*N*=926)

	Could not have said no		Could have said no		Total	
			
Consent categories	*N*	%	*N*	%	*N*	%
Active consent	476	67.0	78	36.1	554	59.8
No objection	113	15.9	112	51.9	225	24.3
Ambivalent	34	4.8	2	0.9	36	3.9
No consent	66	9.3	2	0.9	68	7.3
Indeterminate	21	3.0	22	10.2	43	4.6
Total	710	100.0	216	100.0	926	100.0
Percent of total	76.7		23.3		100.0	

We modelled the associations between categories of consent and covariates, using “Active Consent” as the reference category, and excluding Indeterminate and Ambivalent categories, which accounted for few respondents (Table 3). HIV-positive respondents were significantly less likely to be in the “No Consent” or “No Objection” categories than in “Active Consent” (OR=0.51, CI=0.27–0.99, and OR=0.63, CI= 0.46–0.85, respectively), compared with HIV-negative respondents. Respondents in the second wealth quartile were significantly less likely to be in the “No consent” category (OR=0.47, CI=0.27–0.82) compared to respondents from the poorest wealth quartile. Clients aged 25–34 and 45+ were significantly less likely (OR=0.61, CI=0.49–0.77, and OR=0.56 CI=0.33–0.97 respectively) to be in the “No Objection” than in the “Active Consent” category compared with younger clients.

**Table 3:** Mutually adjusted effect of age, gender, education, wealth, mode of testing, and HIV status on consent Odds Ratios, Confidence Intervals, and *p*-values for the likelihood of being in “No Objection” or “No Consent” categories, compared to Active Consent (reference category)* (*N*=778)**

	No objection				No consent			
		
	OR	95% CI OR		*p*	OR	95% CI OR		*p*
Age group in years								
18–24†								
25–34	0.61	0.49	0.77	<0.001	0.79	0.51	1.22	0.282
35–44	0.73	0.47	1.14	0.170	1.85	0.48	1.50	0.580
45+	0.56	0.33	0.97	0.040	0.92	0.39	2.15	0.840
Gender								
Female†								
Male	1.52	1.01	2.30	0.095	0.68	0.36	1.29	0.237
Education								
No formal education†								
Primary	1.25	0.78	2.00	0.345	1.24	0.79	1.95	0.349
Secondary or more	1.26	0.68	2.32	0.466	0.96	0.53	1.75	0.895
Wealth index								
Lowest†								
Second	1.08	0.81	1.42	0.604	0.47	0.27	0.82	0.008
Third	1.34	0.94	1.90	0.104	0.76	0.46	1.28	0.302
Highest	1.45	0.90	2.35	0.128	0.90	0.49	1.65	0.734
Mode of testing								
Integrated†								
VCT	0.69	0.34	1.42	0.316	0.49	0.12	1.97	0.316
PMTCT	1.38	0.78	2.43	0.27	1.37	0.64	2.91	0.417
HIV status								
HIV negative†								
HIV positive	0.63	0.46	0.85	0.002	0.51	0.27	0.99	0.048
Country								
Burkina	0.40	0.24	0.66	<0.001	1.33	0.53	3.38	0.544
Kenya	0.45	0.26	0.79	0.005	0.85	0.36	2.03	0.721
Malawi	0.30	0.19	0.45	<0.001	0.77	0.34	1.76	0.534
Uganda	0.40	0.24	0.66	<0.001	1.33	0.53	3.38	0.544

* Results are adjusted for clustering at the interview facility level

** excludes respondents classified as Indeterminate or Ambivalent, and those respondents missing information on mode of testing or covariates

† reference category.

### Respondents’ reasons

#### Why 7% respondents tested without consent

Apart from a few respondents who were too sick to give consent, testing without consent occurred when testing was presented as mandatory. This happened in the course of Prevention of Mother-To-Child Transmission (PMTCT), when pregnant women assumed that HIV testing was part of antenatal care and that they had no choice, saying for example: *We were told that testing is a must for pregnant women these days—no testing, no assistance from the doctor;* or *they said if I don’t get tested I won’t continue with antenatal clinic (Malawi);* or *there was no option to refuse unless you do not attend ANC* (Uganda).

Testing was also perceived as a prerequisite to further medical care among those who were ill or hospitalized, as in the following:

*The nurse told me they test all in the ward. (male, Kenya)*

*Provider said the policy […] is for everyone to be tested. (female, Kenya)*

*I thought it was compulsory. (female, Malawi)*

*It is compulsory for anyone who goes to acute. (male, Uganda)*

*It was one of the exams that I had to do before my surgery. (male, Burkina Faso)*

*Providers insisted they absolutely wanted me to test, so I was obliged to do it. (female, Burkina Faso)*

#### Why 77% respondents thought they could not have said no

The reasons invoked by respondents who thought they could not have said no (689, excluding those “Indeterminate”) are shown in Table 4. The main reasons were:

**Table 4:** Reasons why respondents thought they could not refuse HIV testing, by categories of consent and covariates (*N*=689)*

	Reasons why respondents thought they could not say no			
	
Consent category	Decided	Health	Provider	Too late
Active	70.0	29.0	1.1	0
No objection	37.7	11.3	50.0	0.9
Ambivalent	0	23.5	47.1	29.4
No consent	0	1.5	98.5	0
Sex				
Male	52.3	23.7	22.7	1.3
Female	57.9	22.6	17.2	2.3
Age in years				
18–25	55.0	17.0	26.0	2.0
25–34	53.0	22.6	22.6	1.9
35–44	52.1	34.2	12.8	0.9
45+	59.2	30.6	10.2	0
Education				
None	35.5	43.3	21.1	0
Primary	52.5	25.3	21.3	1.0
Secondary+	61.2	15.7	20.4	2.7
Assets quartiles				
Lowest	48.8	26.9	23.8	0.6
Second	58.7	23.8	15.9	1.6
Third	51.6	26.1	21.7	0.6
Highest	55.1	18.0	23.4	3.6
Modes of testing				
Integrated	57.4	24.7	16.6	1.3
VCT	70.8	22.0	4.9	2.4
PMTCT	47.3	21.8	29.1	1.8
HIV status**				
HIV+	51.8	33.3	14.4	0.5
HIV−	55.4	18.9	23.6	2.2
Country				
Burkina	25.0	42.6	26.9	5.6
Kenya	56.9	15.2	26.1	1.7
Malawi	58.6	24.8	16.6	0
Uganda	65.1	20.2	14.0	0.6
Total (*N*=689)	373	161	144	11
Percent	54.1	23.4	20.9	1.6

* Among 710 who thought they could not say no, and excluding 21 respondents classified as indeterminate

** association statistically significant, *p*<0.05

I had decided to test/knowing your status is important (54%)

Just over half of respondents who thought they could not have refused said that they had previously made up their mind and that testing was their choice, with some implying that the question “Could you have said no?” did not make sense. This is illustrated in the following:

*I am the one who decided to go and test. (male, Burkina)*

*If I came here, it is that I really want to test. I don’t see why I would say no. The nurse simply wants to help me know my status and that is what I want. (female, Burkina)*

*That is the purpose that brought me to the facility. (female, Uganda)*

Some in this group said they could not refuse because knowing their HIV status was important:

*It was important to know my status and that of [my] unborn child. (female, Kenya)*

*… because I am preparing for the future of my baby. (female, Malawi)*

*I felt it was important for us. Furthermore, I wanted to build the confidence and trust between me and my boyfriend. (female, Kenya)*

Testing is necessary for treatment and health (23%)

Health concerns were invoked by respondents who perceived testing as necessary to receive care, as in the following:

*Testing is a choice if you seek better health, and therefore refusing would not be good. (female, Burkina)*

*I was coughing a lot and I wanted to know my status [and] what exactly the cough was. (male, Uganda)*

*I wanted treatment that exactly suits my disease, which could be only seen after testing. (male, Uganda)*

*I wanted to know indeed and start medication early. (male, Kenya)*

*I wanted to know if I have the illness or not, and be able to be treated or protect myself. (female, Burkina)*

Women tested in PMTCT said they could not refuse because *you have to test, because of the child, to protect it, or to take precautions [for] my baby*.

Too late to change my mind (2%)

A small percentage of respondents, mostly in the “Ambivalent” category, felt they had previously agreed and could not go back on their decision. A Burkina Faso man explained how, having agreed, one *can no longer refuse [and] must let providers do what they have to do*. A Kenyan man said it was *too late for me to change my mind*. A Ugandan woman said she *had to follow through*, while a Burkina woman said that because she had already agreed to go into the counselling session, she felt obliged to stay and test.

Health providers’ influence (21%)

Respondents who expected providers would care for them thought they should not refuse testing when offered. Several in Burkina Faso expressed their respect for health care providers, asking rhetorically *why would you refuse those who treat you?* Similarly, a Malawi woman said she could not say no *because I know they will help me*. Another woman in Burkina Faso explained that it makes little sense to refuse, because *otherwise health care providers would not be able to treat [you] and care for you, and if you get worse, what would you do?*

The texts describe a range of ways that providers influence decisions: being helpful and friendly, explaining and counselling, encouraging and convincing, and applying various degrees of pressure. For example, a man in Burkina Faso explained that health care providers *told us that they would help us live a little longer, and how could one refuse?* A Malawi woman said: *because of the way I was advised, it was tough to say no*. Some respondents mentioned fear of disapproval. A Burkina woman said: *If you do not test, they don’t look at you right when you go to weigh the baby*. Stronger provider insistence and excessive pressure were experienced by some respondents, as illustrated by the quotes in the section about perceived mandatory testing.

We modelled the reasons given by those who thought they could not have said no, comparing health and provider reasons respectively to “having decided” as the reference category (Supplementary file S3). Invoking provider reasons was not associated with covariates, except that it was less likely for those tested through Voluntary Counselling and Testing (VCT), and more likely for PMTCT. Invoking health reasons was more likely among HIV-positive respondents and those aged 35+; it was less likely among those with education at or beyond primary compared to those with no education.

#### Complex decisions, similar discourse

Respondents’ statements provide insights into the complexity of the decision process, particularly when apparently contradictory points are juxtaposed in the same statement. Several respondents who referred to pressure from providers also noted that testing was for their own good. A Burkina woman said: *if you come to weigh the baby, they tell you to wait at the door [for testing], and the way it is done it is hard to refuse. I think it is important since it is designed to take precautions to protect the baby*. Similarly, a Ugandan woman said: *Because it was [for] my own good, but the provider had also told me I needed the test, so I could not say no*. Even respondents in the “No Consent” category, seemed to appreciate the logic of mandatory testing. For example, a woman from Burkina Faso said: *If you come to the hospital and they ask you [to test] it is hard to refuse because they want [to protect] your health*. A Kenyan woman who did not consent to PMTCT testing said: *When you are expecting, you have to know*. And a Kenyan man who had mandatory testing said: *I wanted the employment, plus it was an opportunity for me to know my status*. Thus even those who explicitly did not consent sometimes acknowledged the benefits of testing, and expressed acceptance or resignation.

There is also a degree of similarity among respondents’ statements, regardless of whether or not they thought they could have said no. Across all countries, and whether or not they thought they could have refused, active consenters referred to individual choice, rights and decisions, as illustrated in Table 5.

**Table 5:** Similarities in the discourse about decisions to test, among those who thought/did not think they could have refused to test (illustrative quotes from four countries)

	Could have refused	Could not have refused
Burkina Faso	*It is a choice. Testing helps people*. *It is better to accept. (M)*	*I was not forced to test, it is my choice. But I could not refuse because it is free and protects the child if I have the illness. (F)* *If I hadn’t wanted to test I wouldn’t have agreed to it. (F)*
Kenya	*It is my right to do so. (F)*	*[…] anyone has the right to say yes or no. (F)*
Malawi	*I wanted to know my status and the illness that I had to be treated. (M)*	*I went voluntarily and wanted to know my status. (M)*
Uganda	*Because I make my own decisions. (F)*	*Because I came on my own and was willing to test. (F)*

## Discussion

Textual analyses show that 7% of 926 respondents had not consented. Had we taken closed-ended responses to the question “Could you have said no?” as indicating lack of consent, as some studies have done, this would have overestimated the percentage of “No consent” at 77%. The 84% frequency of consent we found here (subtotal of Active Consent and No Objection in Table 2) is consistent with our previous finding that 86% of respondents reported a complete consent index [17].

We found that over one quarter of respondents could not be unambiguously classified into consenting or not consenting (24% had “No Objection” and 4% were “Ambivalent”). This is consistent with a careful qualitative analysis of consent in Tanzania, where about one third of 25 respondents indicated neither clear consent nor refusal [20]. Thus, whereas legal or ethical definitions of consent are binary, in reality, there are gradations to consent.

Very few of the associations between categories of consent and socio-economic covariates were statistically significant. This suggests that these categories have relevance across different countries and social groups, and that the likelihood of being in a given consent category was not a function of being in a particular socio-demographic or socio-economic group. The finding that HIV-positive respondents were more likely to be in the “Active Consent” category provides reassurance that they were not at greater risk of coercion to test; we examined whether there were more retesters among those HIV-positive, and found that the proportion of retesters was similar among HIV-positive and HIV-negative respondents (55.6 and 52.3% respectively, *p*=0.753).

When explaining why they said they could not have refused, respondents referred to previous decisions, believing testing was necessary for treatment and health, and the influence of health providers. Health providers’ influence is reported across all modes of testing, and particularly among PMTCT testers. Our results also indicate that among those tested through PMTCT, the percent who actively consented was slightly lower, and the percent who expressed “No Objection” was slightly higher, than among those tested through other modes. But the difference was not statistically significant, and PMTCT testers were not less likely to have consented; and while the statements of women tested through PMTCT do indicate some pressure, they also show that women recognized the importance of testing.

In general, respondents expressed agency even while reporting pressure to test. As argued in a conceptual analysis of voluntariness [11], pressure and constraints are not necessarily incompatible with consent. Nevertheless, continued vigilance is needed to ensure the right to voluntary consent, particularly in view of the possibility of excessive pressure from providers, as documented here in a small proportion of cases.

Our results confirm that offering testing at health facilities does not appear to jeopardize informed consent [17]. The high level of consent we found, compared to studies from earlier years suggests both that services around HIV testing may have improved, and that their acceptability has increased, such that more clients agree to test [21]. The considerable percentage of respondents in the “No Objection” category (24%) also suggests that many individuals are undecided and can be encouraged to test voluntarily when services around testing are friendly, informative and convenient [22].

A literature review on informed consent recommended that researchers probe the responses of respondents in developing countries, who are less likely to refuse, and more likely to worry about consequences than their counterparts in higher income settings [12]. The reports of clients in four countries reveal that perceiving no option to refuse does not automatically indicate coercion, and may, in fact, reflect a considerable degree of agency. “Could you have said no?” is not a good measure of consent, though it is useful to elicit information about the factors that influence consent.

There are limitations to this study, including the use of a facility-based sample that was selected in a systematic rather than random manner, such that respondents are not necessarily representative of the population as a whole or of health care facility users. However, since the goal was to compare measures of consent among those tested at health care facilities, this approach was deemed to be appropriate. The fact that interviews took place at health care facilities may have led respondents to give more favorable answers to questions about health care services. We do not, however, think that such social desirability bias is likely. In a previous analysis, we compared outcomes among respondents who tested at facilities where interviews were conducted and those who reported testing at facilities not included in the MATCH project. We found that responses among those tested at MATCH facilities were similar to, or less positive than, among those who did not test at MATCH facilities. This suggests that the facility environment did not substantially influence respondents’ reports on the care they received; hence, social desirability bias did not have much effect on responses.

Other possible limitations are inherent to qualitative research. When participants are asked to give responses to open-ended questions in their own words, the length and depth of textual responses cannot be standardized, and judgement has to be exercised in recoding. However, such texts are valuable precisely because they represent spontaneous expressions of respondents’ views. In addition, we went to great lengths to consistently recode the texts and standardize the way in which decisions were made about how to categorize responses, such that the possibility of misclassification is negligible. Moreover, unlike much qualitative research, which tends to be small-scale, this study elicited textual responses from over 900 respondents. This provided the opportunity to bring together quantifiable data and texts, and to integrate statistical and textual analyses. We believe our effort to combine quantitative rigor and sensitivity to clients’ views lends credence to the results.

Our results underscore the importance of carefully defining and measuring the three components of informed consent [11]. Information and competence to choose can be operationalized as binary indicators and assessed through closed-ended questions, as was done in our previous analysis [17], but voluntariness is better measured through qualitative methods that elicit respondents’ perceptions of consent as a process. The findings have implications for how consent is conceptualized and measured in diverse settings [11, 12], and, more generally, for international bioethics research on voluntariness [23, 24].

## Conclusions

Textual analyses indicate that 7% of respondents tested without consent, about one quarter expressed no objection to testing, and the majority (60%) actively consented. This is in contrast to the high percentage (77%) who thought they could not refuse. Retrospectively asking clients if they could have refused HIV tests would overestimate coercion. Many individuals are undecided, and, hence, improving the friendliness and quality of services can encourage them to test.

Clients described the influence of various factors, including pressure from providers, but their statements nevertheless indicate voluntariness, thus lending support to efforts to scale-up testing at health facilities.

The analysis also shows that “Could you have said no?” does not measure consent, but can be used to elicit information about reasons for agreeing to test. Triangulating qualitative and quantitative data provides insights into clients’ views and a more accurate measure of consent.

## References

[1] Bayer R, Fairchild AL (2006). Changing the paradigm for HIV testing – the end of exceptionalism. N Engl J Med.

[2] Gruskin S, Ahmed S, Ferguson L (2008). Provider-initiated HIV testing and counselling in health facilities – what does this mean for the health and human rights of pregnant women?. Dev World Bioeth.

[3] Jürgens R (2007). Increasing access to HIV testing and counselling while respecting human rights – background paper.

[4] Obermeyer C. Makhlouf, Osborn M (2007). The uptake of testing and counseling for HIV: a review of the social and behavioral evidence. Am J Public Health.

[5] Baggaley R, Hensen B, Ajose O, Grabbe KL, Wong VJ, Schilsky A (2012). From caution to urgency: the evolution of HIV testing and counselling in Africa. Bull World Health Organ.

[6] Hensen B, Baggaley R, Wong VJ, Grabbe KL, Shaffer N, Lo YR (2012). Universal voluntary HIV testing in antenatal care settings: a review of the contribution of provider-initiated testing & counselling. Trop Med Int Health.

[7] Obermeyer CM, Bott S, Bayer R, Desclaux A, Baggaley R (2013). HIV testing and care in Burkina Faso, Kenya, Malawi and Uganda: ethics on the ground. BMC Int Health Hum Rights.

[8] Angotti N, Dionne KY, Gaydosh L (2011). An offer you can’t refuse? Provider-initiated HIV testing in antenatal clinics in rural Malawi. Health Policy Plan.

[9] Njeru MK, Blystad A, Shayo EH, Nyamongo IK, Fylkesnes K (2011). Practicing provider-initiated HIV testing in high prevalence settings: consent concerns and missed preventive opportunities. BMC Health Serv Res.

[10] Larsson EC, Thorson A, Pariyo G, Conrad P, Arinaitwe M, Kemigisa M (2012). Opt-out HIV testing during antenatal care: experiences of pregnant women in rural Uganda. Health Policy Plan.

[11] Appelbaum PS, Lidz CW, Klitzman R (2009). Voluntariness of Consent to Research: A Conceptual Model. Hastings Center Report.

[12] Mandava A, Pace C, Campbell B, Emanuel E, Grady C (2012). The quality of informed consent: mapping the landscape. A review of empirical data from developing and developed countries. J Med Ethics.

[13] Abdool Karim Q, Abdool Karim SS, Coovadia HM, Susser M (1998). Informed consent for HIV testing in a South African hospital: is it truly informed and truly voluntary?. Am J Public Health.

[14] Weiser SD, Heisler M, Leiter K, Percy-de Korte F, Tlou S, DeMonner S (2006). Routine HIV testing in Botswana: a population-based study on attitudes, practices, and human rights concerns. PLoS Med.

[15] Ujiji OA, Rubenson B, Ilako F, Marrone G, Wamalwa D, Wangalwa G (2011). Is ‘Opt-Out HIV testing’ a real option among pregnant women in rural districts in Kenya?. BMC Public Health.

[16] Guenter D, Barbara AM, Shaul RZ, Yudin MH, Remis RS, King SM (2008). Prenatal HIV testing: women’s experiences of informed consent in Toronto, Ontario. J Obstet Gynaecol Can.

[17] Obermeyer CM, Neuman M, Desclaux A, Wanyenze R, Ky-Zerbo O, Cherutich P (2012). Associations between mode of HIV testing and consent, confidentiality, and referral: a comparative analysis in four African countries. PLoS Med.

[18] QSR (2011). N-Vivo 9.2.

[19] StataCorp (2009). Stata Statistical Software: Release SE 10.1.

[20] Groves AK, Maman S, Msomi S, Makhanya N, Moodley D (2010). The complexity of consent: women’s experiences testing for HIV at an antenatal clinic in Durban, South Africa. AIDS Care.

[21] Rujumba J, Neema S, Tumwine J, Tylleskär T, Heggenhougen K (2013). Pregnant women’s experiences of routine counselling and testing for HIV in Eastern Uganda: a qualitative study. BMC Health Serv Res.

[22] Angotti N, Bula A, Gaydosh L, Kimchi E, Thornton R, Yeatman S (2009). Increasing the acceptability of HIV counselling and testing with three C’s: convenience, confidentiality and credibility. Soc Sci Med.

[23] Marshall P (2006). Informed consent in international health research. J Empir Res Hum Res Ethics.

[24] Kass N, Maman S, Atkinson J (2005). Motivations, understanding and voluntariness in international randomized trials. Hastings Center report. IRB Ethics Hum Res.

